# Survival Strategies and Color Preferences of Mandarin Fish (*Siniperca chuatsi*) and Mud Carp (*Cirrhinus molitorella*): Implications for Aquaculture

**DOI:** 10.3390/ani15040557

**Published:** 2025-02-14

**Authors:** Miao Xiang, Nian Wei, Haoran Liu, Mulan Liao, Zihao Meng, Xuemei Li

**Affiliations:** 1 Yangtze River Fisheries Research Institute, Chinese Academy of Fishery Sciences, Wuhan 430223, China; xiangmiaoihb@163.com (M.X.); weinian@yfi.ac.cn (N.W.); 13697265236@163.com (M.L.); mengzh@yfi.ac.cn (Z.M.); 2 College of Life Science, Huzhou University, Huzhou 313000, China; lhr020620@dingtalk.com

**Keywords:** animal welfare, habitat coloration, fish behavior, predator–prey system, preference

## Abstract

This study evaluated the habitat coloration preferences of *Siniperca chuatsi* and *Cirrhinus molitorella* in both solitary (*n* = 1) and group (*n* = 3) settings across six colors. The results indicated that both individual and group *S. chuatsi* spent the majority of their time in and made frequent visits to the black area, followed by the blue area. While individual *C. molitorella* spent the majority of their time and visits in the blue region, groups showed a preference for the blue and white regions. These findings highlight the distinct habitat coloration preferences of *S. chuatsi* and *C. molitorella* in different group states, suggesting that habitat coloration has an important effect on fish behavior and environmental adaptation. From a behavioral ecology perspective, these preferences may be closely related to the survival strategies of fish. Notably, *C. molitorella*’s strong preference for blue and white backgrounds may reflect anti-predator behavior, helping *C. molitorella* avoid predators, such as *S. chuatsi*, in natural environments, thereby improving its chances of survival. This study provides a scientific basis for optimizing aquaculture environments, emphasizing the importance of considering habitat coloration and substrate type in designing environments to enhance fish welfare.

## Simple Summary

This study aimed to investigate the habitat coloration preferences of *Siniperca chuatsi* (a predator) and *Cirrhinus molitorella* (a prey). The purpose of this study was to examine the influence of habitat coloration on fish behavior, so as to improve animal welfare in aquaculture as well. The results revealed that *S. chuatsi* preferred black and blue backgrounds, while *C. molitorella* showed a preference for blue and white backgrounds. Divergent habitat coloration preferences between *S. chuatsi* and *C. molitorella* were identified as indicating an anti-predator strategy of *C. molitorella*, potentially aiding in predator evasion in natural habitats. Consistent habitat coloration preferences might reflect camouflage behavior in *S. chuatsi*. These findings contribute to optimizing aquaculture environments to enhance fish health and welfare while offering new insights into behavioral adaptations in fish.

## 1. Introduction

The study of animal welfare has gained increased attention in recent years from scholars and researchers across various fields of enquiry [1]. Specifically, animal welfare is defined as the ability of animals to adapt to their environment and satisfy their basic natural needs. This concept encompasses various aspects, including animal health, comfort, nutrition, safety, and the ability to express natural behaviors without experiencing pain, fear, or stress [2,3,4]. The living environment, husbandry practices, and management techniques employed in aquaculture settings can directly or indirectly compromise the mental and physical well-being of animals [5,6,7]. For example, changes in the aquatic environment can trigger stress responses in animals, which in turn affect their natural behavior and reduce aquaculture production [8,9]. The challenge of accurately measuring the emotions of aquaculture animals is well documented [10]; however, the suitability of living environments can be assessed through behavioral changes [11]. Therefore, observing and understanding the behavioral patterns of aquaculture animals is crucial for improving their welfare [2,3,4]. The welfare of fish, as an important farmed species, is of particular significance. Fish possess perceptual abilities, including the capacity to make choices, experience fear-like states, recognize conspecifics, and actively avoid unfavorable conditions [3,11]. However, introducing new habitats or altering their natural habitats can negatively affect fish welfare in aquaculture practices. Artificial environments such as tanks, nets, and ponds can negatively impact the feeding activities, growth, health, and welfare of cultured fish under stressful conditions [12]. Nevertheless, from an economic standpoint and in the commercial interest of clean, healthy farming, fish farmers typically prioritize replicating natural habitats or creating better environments for fish [13]. Although studies have been conducted to explore the relationship between fish behavior and environmental preferences as well as aquaculture welfare, there is still a lack of research on the background color preferences of *S. chuatsi* and *C. molitorella* in different population states. *S. chuatsi*, as an economically important fish, has a predatory behavior and survival strategy that is closely related to the background color. The preference of *C. molitorella*, as the natural bait of *S. chuatsi*, in terms of the background color may affect its survival chances in the natural environment. In this study, experimental observations revealed the preferences of *S. chuatsi* and *C. molitorella* for different background colors under individual and group conditions, providing new perspectives for understanding the behavioral adaptations of the two species and shedding light on the culture welfare of these two species.

The mandarin fish (*Siniperca chuatsi*) is an economically significant freshwater-cultured species in China [14]. This carnivorous species primarily preys on smaller fish throughout its life and plays a key role in maintaining ecosystem stability [15]. The mud carp (*Cirrhinus molitorella*), a common omnivorous freshwater fish, and the *C. molitorella*, known for its swift growth and strong adaptability, serve as the perfect prey for the *S. chuatsi* [16,17,18,19]. This predator–prey relationship is essential for the growth and health of *S. chuatsi* and holds significant value for the study of fish behavior and ecology. The adaptive mechanisms of *S. chuatsi* and *C. molitorella* in natural and artificial environments are critical to ecosystem stability. They form predator–prey relationships that affect not only their own survival and health, but also the functioning of the whole ecosystem. An in-depth study of these mechanisms can help to understand their ecological roles and provide theoretical support for optimizing the aquaculture environment. Factors such as the habitat coloration preference in the aquaculture environment of *S. chuatsi* may influence its welfare [20]. It is well established that different fish species exhibit divergent preferences for habitat coloration, which are associated with their survival strategies and visual perception [21]. For instance, zippered David’s Schizothoracin (*Schizothorax davidi*) shows variation in color and substrate preference based on group size. Studying the habitat coloration preference of carnivorous fish can help to achieve a more comprehensive understanding of fish behavior, thereby enabling the optimization of aquaculture environments and enhancing fish welfare [22,23,24]. Moreover, the observation that *Pelteobagrus fulvidraco*, which exhibits distinct personality traits, may share a similar preference for color selection offers novel insights into the behavioral and welfare dynamics of fish [22,25]. Additionally, the potential to differentiate between mandarin fish and mud carp in terms of habitat coloration selection warrants further exploration.

The present study offers insights into the behavior of *S. chuatsi* and *C. molitorella* regarding their habitat coloration preferences under both individual and group aquaculture conditions. Experimental observations were conducted under six different habitat coloration (red, yellow, blue, green, black, and white). The behavioral performance of these two fish species was analyzed in detail under individual and group conditions. The scientific value of examining individual fish behavior lies in elucidating the mechanisms underlying these behaviors, thereby establishing a theoretical foundation for enhancing the culture environment, optimizing culture efficiency, and promoting fish welfare. While fish typically inhabit groups in natural habitats, studying individual behavior offers the advantage of discerning individual variations and identifying their true preferences. This approach enables precise guidance for aquaculture management and circumvents the challenges posed by group behavior, which can compromise the reliability of experimental outcomes. The experiment employs a simulation of the transition state from low to high density by studying individual and small-group behaviors, thereby revealing the effects of density changes on behavior and providing a theoretical foundation for optimizing culture conditions. This study may provide valuable insights for the aquaculture industry in terms of improving fish welfare and adaptation by adjusting habitat coloration, which can enhance aquaculture efficiency and fish health.

## 2. Materials and Methods

### 2.1. Fish Maintaince

*S. chuatsi* and *C. molitorella* were hatched in the same year using artificial breeding techniques at the Yangtze River Fisheries Research Institute, Chinese Academy of Fisheries Sciences. The two species were then reared separately in an indoor recirculating aquaculture system. During the feeding period, *S. chuatsi* was fed with *C. molitorella*, while *C. molitorella* was fed commercial feed at 8:00 a.m. Prior to formal experiments, the experimental fish were starved for 24 h. The standard length and body weight of the two test species were measured (Table 1). The water source was pond water, which was filtered and aerated in a reservoir for 48 h. Since colors are caused by the reflection of light, if we conduct experiments at night, these colors are reflected as black and the background does not show the corresponding color. There is no need to conduct the experiment at night in order to ensure the accuracy of the content of the study. Therefore, the experiment was conducted from 8:00 a.m. to 6:00 p.m. every day, ensuring that no anthropogenic noise was present during the experimental period. After each trial, the water was replaced to eliminate any residual odors from the fish. Although the water was not changed frequently during the actual breeding process, in the behavioral experiments, the water was changed when each set of replicates was carried out in order to avoid the mucus or odor of the previous fish influencing the selection of the next test fish and to ensure, as far as possible, that the only variable in the experiment was the choice of the background color [22,24]. The mean water temperature was recorded as 18 ± 0.5 °C, the mean dissolved oxygen concentration was 8.0 ± 0.5 mg/L, and the pH was 7.9 ± 0.2. Neither water changes nor aeration were performed during the experimental period. The light cycle consisted of 11 h of light and 13 h of darkness, with light intensity ranging from 0 to 500 lux, except during the feeding period [26]. A running-water system was used in the experimental design, the duration of each experiment was relatively short (20 min), and a thorough water change operation was carried out at the end of each experiment. No toxic physicochemical indicators, such as ammonia and nitrite, were discovered in the behavioral experiment, and what issues existed were not sufficient to have a significant impact on the behavior of the fish [22,24].

### 2.2. Experimental Setup

**Figure 1:**
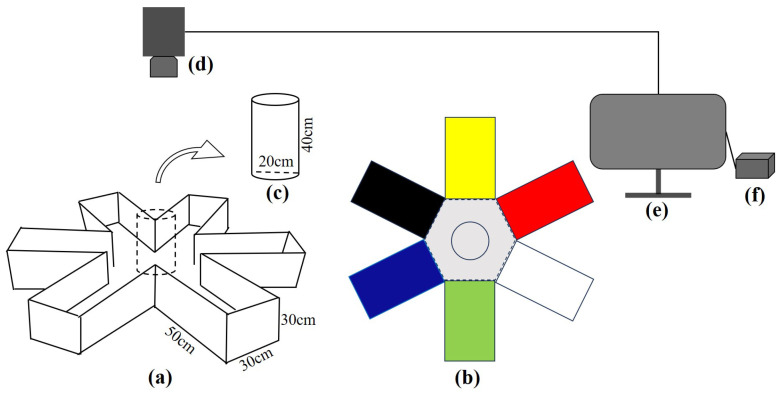
Test tank and behavioral capture system for habitat coloration preference: (**a**) test tank, (**b**) test tank with colors (top view), (**c**) opaque gray cylindrical shield, (**d**) infrared camera, (**e**) monitor, and (**f**) video recorder.

The experimental setup consisted of a radial 6-arm maze (Figure 1). Each arm of the maze was a rectangular structure, measuring 30 cm in length and 50 cm in width. In the center, there was a gray square hexagon with a side length of 30 cm. The water depth was maintained at 20 cm throughout the experiments. Waterproof stickers were sequentially applied inside the radial arms, including red (RGB: 255, 0, 0), yellow (RGB: 255, 255, 0), blue (RGB: 0, 0, 255), green (RGB: 0, 255, 0), black (RGB: 0, 0, 0), and white (RGB: 255, 255, 255) stickers. The more contrasting colors were affixed on the two opposite arms on the device [27].

The maze setup included a 6-arm maze, along with a circular ambient water body (radius 100 cm, water depth 7 cm) to maintain the temperature of the experimental water body. A camera (Sony AX60, 4K, Sony AX60 isSony Corporation, Tokyo, Japan) was positioned directly above the maze. The observation system consisted of a night-vision infrared webcam (Hikvision, DS-2CD864, Hikvision, Hangzhou, China) and a monitor (Hikvision, DS-8832N-R8, Hikvision, Hangzhou, China). The webcam was positioned 2 m above the water surface [28,29].

### 2.3. Experimental Methods

*S. chuatsi* and *C. molitorella* were placed in both individual and group (*n* = 3) settings to enable a comparative analysis of their behavioral performance across different habitat colorations. A setup was used in which four groups of fish were assessed independently, and observations were made on individual fish and groups of fish by repeating the experiment, using a total of six different habitat colors: red, yellow, blue, green, black and white. This approach was adopted to gain a comprehensive understanding of fish preferences for various colors.

Before the experiment, the fish were fasted for 24 h to ensure optimal activity levels and sensitivity to the surrounding environment. We set up a 5 min adaptation period, followed by a 20 min experimental shot [22,24]. During the experiment, the fish were placed into the center of the tank to acclimate to the experimental environment for five minutes. Then, the shield was removed and timer was started to record the residence time and number of visits of the fish to different areas of the tank for 20 min. The experimental environment was kept quiet during the experiment to avoid external interference. The behaviors of the fish were recorded in real time using the observation system. The behavioral data were processed by calculating the percentage of time spent and the visits of experimental fish under each habitat coloration.

### 2.4. Data Analysis

In behavioral choice experiments, the percentage of dwell time and number of visits are widely used as selection metrics. This ensures the accuracy of the data due to the high number of repetitions in each group experiment. However, in group experiments, due to the limitations of the automated tracking software, we used manual observation to analyze the time and number of visits, relying on observation only. This is an intuitive indicator of fish selection trends. The dwell time and visit frequency of each individual (both as a singleton and in a group) in different habitat colorations were recorded over 20 min. The stay time and frequency of fish in different habitat colorations were calculated as follows [24].

*P_t_* = *t*/*T* × 100

where *P_t_* is the percentage of cumulative dwell time (%) of the experimental fish under different colors, *t* is the cumulative dwell time per fish under each color, and *T* is the total cumulative dwell time per fish across all six colors.

*P_f_* = *f*/*F* × 100

where *P_f_* is the percentage of visit frequency (%) of experimental fish under each color, *f* is the visit frequency per fish under each color, and *F* is the total visit frequency per fish across all six colors. For the group trials, we calculated the average dwell time and visit frequency by dividing by the number of individuals.

All data were initially tested for normality and homogeneity of variance using the Kolmogorov–Smirnov and Levene tests. The Kruskal–Wallis test was then applied to evaluate differences in the behaviors of the experimental fish under different habitat colorations. All data were presented as the mean ± S.D., and the significance threshold was set at *p* < 0.05.

## 3. Results

### 3.1. Preference of Individual Siniperca Chuatsi and Cirrhinus Molitorella

**Figure 2:**
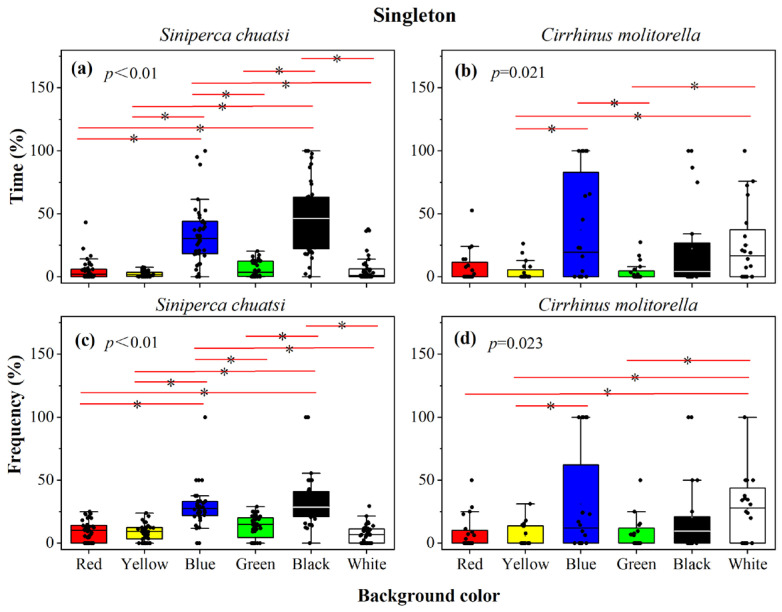
The percentage time (**a**,**b**) and frequency (**c**,**d**) of individuals of *Siniperca chuatsi* and *Cirrhinus molitorella* in different habitat colorations. Note: * indicates significance. The same is seen below.

In the individual group, there was a significant difference in the percentage of time *S. chuatsi* spent in each color area (Kruskal–Wallis tests, *p* < 0.01; Figure 2a, Table A1). A total of 40 sets of replicates were performed to ensure the accuracy of the experiment. *S. chuatsi* spent the most time in the black area (47.10 ± 4.39%), followed by the blue (33.78 ± 3.70%), green (5.96 ± 0.97%), white (5.85 ± 1.63%), red (5.08 ± 1.27%), and the yellow (2.24 ± 0.36%) areas. Post hoc comparisons revealed that *S. chuatsi* spent significantly more time in the black and blue areas compared to the other four colors (*p* < 0.05), with no significant difference among the red, yellow, green, and white areas (*p* > 0.05) (Figure 2a, Table A1).The percentage of visits by individual *S. chuatsi* to each color area followed a similar pattern to the dwell time, with black (33.01 ± 3.18%), blue (28.35 ± 2.51%), green (13.37 ± 1.42%), red (9.54 ± 1.28%), yellow (8.74 ± 1.01%), and white (6.99 ± 1.12%) showing the same trend (Kruskal–Wallis tests, *p* < 0.01; Figure 2c, Table A1). Similar to dwell time, *S. chuatsi* visited the black and blue areas significantly more frequently than the other four colors (*p* < 0.05), while the number of visits to the red, yellow, green, and white areas did not differ significantly (*p* > 0.05; Figure 2c, Table A1).

For *C. molitorella*, there was a significant difference in the percentage of time spent in each color region (Kruskal–Wallis tests, *p* = 0.021; Figure 2b, Table A1). A total of 20 sets of replicates were performed to ensure the accuracy of the experiment. *C. molitorella* spent the most time in the blue region (37.07 ± 9.50%), followed by white (25.17 ± 6.80%), black (22.33 ± 8.10%), red (7.59 ± 2.94%), green (3.93 ± 1.64%), and yellow (3.91 ± 1.66%) areas in that order. Post hoc analysis revealed that *C. molitorella* spent significantly more time in the blue and white areas compared to yellow and green locations (*p* < 0.05), with no significant difference in residence time among the remaining colors (*p* > 0.05; Figure 2b, Table A1). Regarding the number of visits, *C. molitorella* showed a significant difference in the percentage of visits across color regions (Kruskal–Wallis tests, *p* = 0.023; Figure 2d, Table A1). The pattern of visit percentages was similar to the temporal pattern: blue (37.07 ± 9.50%), white (27.07 ± 5.83%), black (20.56 ± 6.94%), red (8.19 ± 3.01%), green (7.53 ± 2.77%), and yellow (5.71 ± 2.01%). Post hoc analysis revealed that the number of visits to the white area was significantly higher than to the red, yellow, and green areas (*p* < 0.05), and there were significantly more visits to the blue area than to the yellow area (*p* < 0.05). No significant differences were found among the remaining groups (*p* > 0.05; Figure 2d, Table A1).

### 3.2. Preference of Siniperca Chuatsi and Cirrhinus Molitorella

**Figure 3:**
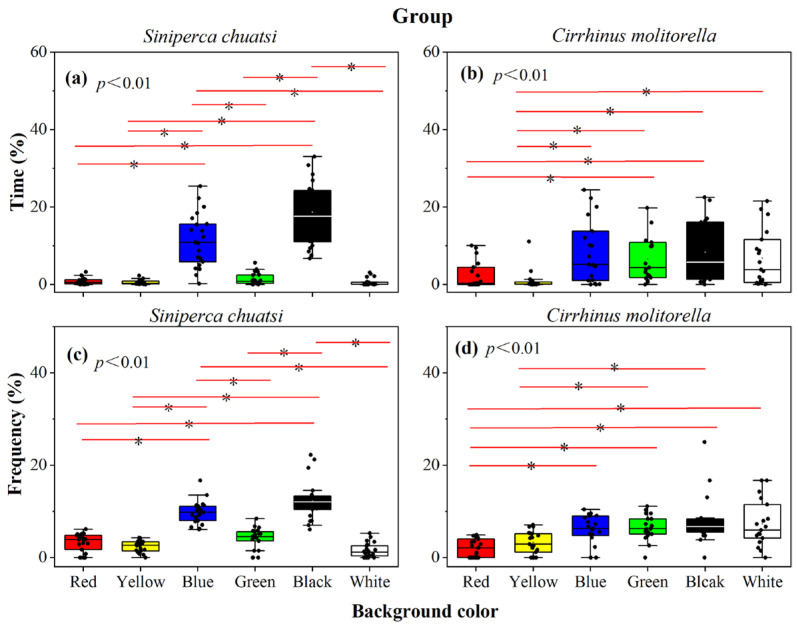
The percentage time (**a**,**b**) and frequency (**c**,**d**) of groups of *Siniperca chuatsi* and *Cirrhinus molitorella* in different habitat colorations.

A significant difference was observed in the percentage of time spent by *S. chuatsi* in each color area in the population group (Kruskal–Wallis tests, *p* < 0.01; Figure 3a, Table A1). A total of 21 sets of replicates were performed to ensure the accuracy of the experiment. The black area accounted for the longest percentage of time spent (18.63 ± 1.77%), followed by the blue (11.42 ± 1.51%), green (1.36 ± 0.34%), red (0.80 ± 0.18%), white (0.59 ± 0.21%), and yellow (0.54 ± 0.13%) areas. Post hoc analysis revealed that *S. chuatsi* groups spent significantly more time in the black and blue areas compared to the other four colors (*p* < 0.05), while no significant differences were found in the red, yellow, green, and white areas (*p* > 0.05, Figure 3a, Table A1).The percentage of visits to each color area by *S. chuatsi* groups was as follows: black (12.32 ± 0.93%), blue (9.80 ± 0.55%), green (4.11 ± 0.51%), red (3.21 ± 0.44%), yellow (2.37 ± 0.27%), and white (1.53 ± 0.34%). This was the same pattern as seen with the differences in the duration of time spent in each color zone (Kruskal–Wallis tests, *p* < 0.01; Figure 3c, Table A1). Similarly, *S. chuatsi* populations exhibited a significantly higher percentage of visits to the black and blue areas compared to other four colors (*p* < 0.05), while no significant differences were observed in the number of visits between red, yellow, green, and white areas (*p* > 0.05; Figure 3c, Table A1).

A significant difference was observed in the percentage of residence time in each color region for the *C. molitorella* population group (Kruskal–Wallis tests, *p* < 0.01; Figure 3b, Table A1). A total of 19 sets of replicates were performed to ensure the accuracy of the experiment. The longest percentage of time was spent in the black area (8.28 ± 1.77%), followed by blue (8.28 ± 1.86%), white (6.77 ± 1.63%), green (6.61 ± 1.30%), red (2.37 ± 0.79%), and yellow areas (1.03 ± 0.59%). Post hoc analyses revealed that the dwell time of *C. molitorella* groups was significantly higher in the red area than in the green and black areas (*p* < 0.05), while no significant differences were found with the other colors (*p* > 0.05). The dwell time in the yellow area was significantly lower than that in the blue, green, black, and white areas (*p* < 0.05), with no significant differences observed among the remaining colors (*p* > 0.05; Figure 3b, Table A1). In terms of the frequency of visits, a significant difference was observed in *C. molitorella* groups in each color region (Kruskal–Wallis tests, *p* < 0.01; Figure 3d, Table A1). The percentage of visits to each region followed a certain order: black (8.07 ± 1.23%), white (7.32 ± 1.13%), green (6.48 ± 0.62%), blue (6.16 ± 0.69%), yellow (3.27 ± 0.55%), and red (2.03 ± 0.44%). Post hoc analysis revealed that the number of visits to the red region was significantly lower than that to the blue, green, black, and white regions (*p* < 0.05), and no significant difference was found regarding the visits to the yellow region (*p* > 0.05). The percentage of visits to the yellow region was significantly lower than that to the green and black regions (*p* < 0.05), and no significant differences were observed among the other regions (*p* > 0.05, Figure 3d, Table A1).

## 4. Discussion

### 4.1. Habitat Coloration Selection of Siniperca Chuatsi

The findings revealed that *S. chuatsi*, both as individuals and groups, had advantages in the proportion of dwell time spent in black and blue areas and frequency of visits. Combined with dwell time and visit frequency, these results suggest that *S. chuatsi* prefers a blue and black habitat coloration. These findings reveal a strong preference for these two colors in *S. chuatsi*, which may be closely related to their survival strategies [30].

From the behavioral ecology perspective, this preference may be related to the camouflage behavior of *S. chuatsi*. When the habitat coloration of *S. chuatsi* is consistent, it can be hypothesized that this represents an adaptive camouflage behavior, helping these fish to hide more effectively in their natural environment and gain protection from predators [31,32]. A darker background offers optimal camouflage conditions for *S. chuatsi*, allowing it to conceal itself more effectively during predation or encounters with natural enemies [33]. The blue background may align with the characteristics of *S. chuatsi*’s natural habitat, such as clear waters [34], which provide a sense of security and may also aid in navigation and communication in complex environments [35]. Furthermore, group behavior significantly influenced the habitat coloration selection of *S. chuatsi*. In groups, *S. chuatsi* has been shown to identify and utilize environmental resources more effectively through social interaction and information exchange [36]. The behavior of individuals within a group can be influenced by their peers, resulting in greater consistency in habitat coloration selection [37]. This phenomenon is theorized to enhance the survival chances of individuals within a group, while also augmenting the overall adaptive capacity of the group [38]. Future studies could further investigate the role of visual perception mechanisms in habitat coloration selection in *S. chuatsi*, for example, by examining the effects of different wavelengths of light on the visual system of *S. chuatsi*, and how other environmental factors, such as light intensity and water transparency, interact to affect visual perception and habitat coloration preference in fish. Through these studies, we can gain a more comprehensive understanding of the behavioral adaptations of *S. chuatsi* and provide a scientific basis for optimizing aquaculture environments and improving aquaculture welfare.

### 4.2. Habitat Coloration Selection of Cirrhinus Molitorella

This study provides insights into the habitat coloration preferences of *C. molitorella* at different group sizes. The results showed that individual *C. molitorella* spent the most time in areas with a blue background, followed by a white background. In contrast, groups of *C. molitorella* exhibited a marked preference for black and blue areas. Meanwhile, based on the visit frequency, individuals also showed a clear preference for blue and white areas, while groups preferred black and white, respectively. The group demonstrated the highest stay time and frequency of visits in the black area, indicating its preference was strongest for this color. These results reveal a clear preference for blue backgrounds in *C. molitorella*, with a marked difference between individual and group states. From the perspective of behavioral ecology, this preference may be closely related to *C. molitorella*’s perception of its environment and exploratory behavior [39,40]. The blue background may provide *C. molitorella* with richer visual information, aiding in recognition and enabling more effective utilization of environmental resources.

In group trials, *C. molitorella* demonstrates an enhanced ability to identify and utilize environmental resources through social interactions and information exchange. This social structure may guide individual *C. molitorella* to select a habitat coloration consistent with the group, thereby enhancing both individual survival chances and the group’s adaptive capacity to environmental changes. Notably, according to the habits of *C. molitorella*, *C. molitorella* tends to live in the lower and middle layers of the water column, and tends to move towards brighter areas in order to avoid predation. Insofar as *S. chuatsi* and *C. molitorella* diverge in habitat coloration preference, it may indicate that *C. molitorella* adopts an anti-predator strategy [3,11,41]. This strategy may help *C. molitorella* to avoid predators more effectively in its natural environment [22,26], thereby increasing its survival probability. This divergence in habitat coloration preference can be considered a behavioral adaptation to predation pressure, with ecologically significant and potentially far-reaching implications for survival and reproduction in ecosystems [42,43,44,45,46].

### 4.3. Future Consideration

Despite the valuable insights gained from the present study on the habitat coloration preferences of *S. chuatsi* and *C. molitorella* at both individual and group levels, several limitations and unanswered questions remain. These findings lay a foundation for future research. The study revealed that *S. chuatsi* (a predator) preferred black and blue backgrounds, while *C. molitorella* (a prey) preferred blue and white backgrounds. This divergence in preference may be indicative of an anti-predator behavior in *C. molitorella*, helping it evade predators in its natural habitat [22,26,41]. Simulating experimental conditions that more closely resemble the natural environment could provide a more comprehensive understanding of fish behavioral adaptations in complex environments [5,6,7].

Additionally, the present study could be expanded to include other fish species to compare similarities and differences in habitat coloration selection across species. This approach will help in identifying general trends and special adaptations related to habitat coloration selection in fish. Furthermore, the role of fish visual perception mechanisms in habitat coloration selection should be investigated. Long-term behavioral observations should be conducted to study the behavioral patterns and adaptive changes in fish under various habitat coloration conditions. Changes in behavioral habits, physiological indices, and survival ability of fish after long-term exposure to a specific habitat coloration should be observed in order to understand the adaptive processes and mechanisms of fish to the environment [44]. Finally, the ecological significance of fish habitat coloration choice should be explored, including its impact on population structure, habitat selection, and ecological niche differentiation. The research findings will be applied to aquaculture practice to optimize habitat coloration in aquaculture environments, improving fish growth rate, reproductive success, and the aquaculture welfare of fish. This will provide scientific and technical support for the sustainable development of the aquaculture industry. The in-depth exploration of these future research directions will enhance our understanding of the behavioral mechanisms and ecological significance of fish habitat coloration choice, contributing to the protection of aquatic ecosystems and improving aquaculture benefits.

## 5. Conclusions

This study investigated the habitat coloration preferences of individuals and groups of *S. chuatsi* and *C. molitorella*. *S. chuatsi* showed a preference for black and blue backgrounds, regardless of whether they were alone or in groups. In contrast, solitary *C. molitorella* exhibited the longest dwell time and highest visit frequency on the blue background, followed by the white background. Grouped *C. molitorella* spent more time in the black and blue regions and visited the black and white areas more frequently, indicating varying preferences based on group state. These findings suggest that habitat coloration plays a significant role in influencing fish behavior and environmental adaptation. From a behavioral ecology perspective, these preferences are linked to survival strategies. The influence of group behavior on habitat coloration selection is a key factor; within groups, individuals can more efficiently identify and exploit environmental resources through social interaction and information sharing. The strong preference of *C. molitorella* for blue and white backgrounds may reflect an anti-predator strategy, helping it evade predators like *S. chuatsi* in its natural habitat and thereby increasing its chances of survival. Future research should explore the specific role of visual perception mechanisms in habitat coloration selection and investigate how environmental factors such as light intensity and water clarity influence these preferences, thereby providing a more comprehensive understanding of fish behavioral adaptations. Meanwhile, the discoveries made with this experimental method can be applied to other species or natural environments in the future.

## Figures and Tables

**Table 1:** Standard length and weight of *Siniperca chuatsi* and *Cirrhinus molitorella* across different trials.

Fish		Number of Trials	Total Number	Standard Length/cm	Weight/g
*Siniperca chuatsi*	Singleton	40	40	18.8 ± 1.8	173.9 ± 49.4
	Group	21	63	18.5 ± 1.8	166.4 ± 46.6
*Cirrhinus molitorella*	Singleton	20	20	4.9 ± 0.8	1.7 ± 1.0
	Group	19	57	5.4 ± 1.1	2.5 ± 1.5

## Data Availability

The original contributions presented in the study are included in the article, further inquiries can be directed to the corresponding author.

## Appendix A

**Table A1:** Stay time (%) and visit frequency (%) for both singleton and group *Siniperca chuatsi* and *Cirrhinus molitorella* under six background colors.

Species			Red	Yellow	Blue	Green	Black	White
Time (%)	*Siniperca chuatsi*	Singleton	5.08 ± 1.27%	2.24 ± 0.36%	33.78 ± 3.70%	5.96 ± 0.97%	47.10 ± 4.39%	5.85 ± 1.63%
		Group	0.80 ± 0.18%	0.54 ± 0.13%	11.42 ± 1.51%	1.36 ± 0.34%	18.63 ± 1.77%	0.59 ± 0.21%
	*Cirrhinus molitorella*	Singleton	7.59 ± 2.94%	3.91 ± 1.66%	37.07 ± 9.50%	3.93 ± 1.64%	22.33 ± 8.10%	25.17 ± 6.80%
		Group	2.37 ± 0.79%	1.03 ± 0.59%	8.28 ± 1.86%	6.61 ± 1.30%	8.28 ± 1.77%	6.77 ± 1.63%
Visit frequency (%)	*Siniperca chuatsi*	Singleton	9.54 ± 1.28%	8.74 ± 1.01%	28.35 ± 2.51%	13.37 ± 1.42%	33.0 1± 3.18%	6.99 ± 1.12%
		Group	3.21 ± 0.44%	2.37 ± 0.27%	9.80 ± 0.55%	4.11 ± 0.51%	12.32 ± 0.93%	1.53 ± 0.34%
	*Cirrhinus molitorella*	Singleton	8.19 ± 3.01%	5.71 ± 2.01%	37.07 ± 9.50%	7.53 ± 2.77%	20.56 ± 6.94%	27.07 ± 5.83%
		Group	2.03 ± 0.44%	3.27 ± 0.55%	6.16 ± 0.69%	6.48 ± 0.62%	8.07 ± 1.23%	7.32 ± 1.13%
